# Is There any Alternative Receptor for SARS-CoV-2?

**DOI:** 10.22074/cellj.2021.7977

**Published:** 2021-05-26

**Authors:** Mahtab Shahriari Felordi, Arash Memarnejadian, Mustapha Najimi, Massoud Vosough

**Affiliations:** 1 Department of Regenerative Medicine, Cell Science Research Center, Royan Institute for Stem Cell Biology and Technology, ACECR, Tehran, Iran; 2 Sernova Corp., London, Ontario, Canada; 3 Laboratory of Pediatric Hepatology and Cell Therapy, Institute of Experimental and Clinical Research (IREC), Université Catholique de Louvain, Brussels, Belgium

**Keywords:** Angiotensin-Converting Enzyme II, COVID-19, Endoplasmic Reticulum Stress, HSPA5, SARS-CoV-2

## Abstract

Angiotensin-converting enzyme II (ACE2) in association with type II transmembrane serine protease (TMPRSS2) is considered the main receptor of SARS-CoV-2. However, considering the clinical complications of COVID-19 in different organs, there is no strong association between the abundance of ACE2/TMPRSS2 co-expression and clinical features of the disease and the severity of complications. Since SARS-CoV-2 affects certain organs that lack or have low expression of ACE2/TMPRSS2, it may be possible that the virus employs other receptors for colonization and entry. Based on recent studies, glucose-regulated protein 78 (GRP78) can be a potential alternative receptor for SARS-CoV-2 entry. In this letter, supporting evidence proposed GRP78 as an alternative receptor in SARS-CoV-2 infection.

## Virus clonization and pandemics

World Health Organization declared a global public health emergency of international concern, on 30^th^ January 2020 due to the ongoing pandemic of coronavirus disease 2019 (COVID-19) (1, 2). Clinical features of this disease are extending from an asymptomatic infection to acute respiratory distress syndrome, and multi-organ failure in some cases (2-5). Virus entry and replication relies on a fine interaction between the virus and host cells (6). Studies have shown that the main receptor for SARS-CoV-2 binding is angiotensin-converting enzyme II (ACE2) (7, 8). Successful entry of the virus into host cells depends on two consecutive steps, i. Attachment of the virus to the ACE2 receptor and, ii. Simultaneous activation of type II transmembrane serine protease TMPRSS2 which cleaves and activates the virus spike (S) protein (9-11).

## ACE2 expression in different tissues and COVID-19 pathogenesis

According to the Human Protein Atlas, the ACE2 receptor is abundantly expressed in the gut, kidneys, and testis, and at lower levels in the lungs and heart (12). However, the lungs and heart have been documented as important targets for SARS-CoV2 infection. Furthermore, co-expression pattern of ACE2/TMPRSS2 through the tissues does not explain clinical complications or their severity in COVID-19 patients (13, 14). In addition, SARS-CoV-2 infects organs that lack ACE2, probably through interactions with other receptors. Endocrine cells in the prostate gland, astrocytes and pericytes in the central nervous system, and hepatocytes in liver are examples of cells that do not express ACE2 (15). The expression pattern of ACE2 in the mentioned organs are different from higher levels in male gonads to lower levels in heart and CNS. In the other words, SARS-CoV-2 can cause multi-organ failure and there is no strict correlation between the abundancy of ACE2 and clinical complications.

## GRP78 as a receptor for different viruses

**Fig.1:**
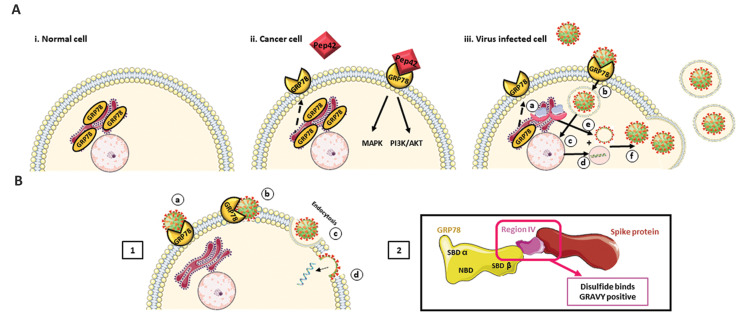
GRP78 in different conditions. **A.** i. Normal cells. GRP78 is an important chaperone in endoplasmic reticulum. ii. Cancer cells. In cancerous cells, GRP78 is translocated to the cell membrane and comprises as a receptor. The main ligand for CS-GRP78 is Pep42 that activates certain pathways at the down-stream and initiate cancerous phenotypes. iii. Virus-infected Cells. GRP78 translocated to the cell surface. CS-GRP78 as a receptor at the cell surface facilitates viral entry into the cell and amplification and release of new viral generations from the host cell. **B.** 1. The proposed mechanism of virus entry through GRP78 receptor. 2. The required energy for virus entry provided by the ABD. CS-GRP78 can interconnect with S protein of SARS-CoV-2 by its SBDβ domain through the constituted disulfide and hydrophobic bonds. ABD; ATP binding domain, CS-GRP78; Cell surface glucose regulated protein 78, NBD; Nucleotide binding domain, SARS-CoV-2; Severe acute respiratory syndrome coronavirus 2, and SBDβ; Substrate binding domain β.

Glucose-regulated protein 78 (GRP78) is used by different viruses for entry into host cells (16, 17). This receptor (also called BiP and HSPA5) is a member of the heat shock protein 70 (HSP-70) family and a master chaperone protein localized on the endoplasmic reticulum (ER) membrane (18). This protein is broadly expressed in many tissues and composed of two structural domains: i. Nucleotide-binding domain (NBD), or ATP-binding domain (ABD) at the N-terminal and, ii. A substrate binding domain (SBD) at the C-terminal (19). The β region of SBD can play a crucial role in facilitating the interaction between protein ligands and the target cell membrane (20). As a response to ER stress, GRP78 overexpresses and translocates to the cell surface. Cell surface GRP78 (CS-GRP78), along with its SBD domain, can act as a multifunctional receptor and recognize various proteins, ligands, and viruses (16). It was shown that cancer cells overexpress CS-GRP78, which is specifically recognized by Pep42, a seven-residue cyclic peptide (21). The motif generated by disulfide bonds in Pep42 can interact with CS-GRP78 (20-22). The cyclic structure of Pep42 stabilizes a hydrophobic motif which strenghtens its affinity to CS-GRP78-SBDβ (20). Molecular modeling and docking analyses have revealed 13 regions which are crucial for disulfide bond formation in the SARS-CoV-2 spike (S) protein. The four disulfide bonds located on the outer surface of the S protein can interact with other ligands. The pairwise sequence alignments and hydrophobicity index comparison between S protein regions and Pep42 revealed a remarkable similarity between the region IV of S protein and Pep42. Considering the fact that Pep42 and GRP78 interact strongly, the structural/biochemical similarity between S protein and Pep42 also suggest that GRP78 can bind to the S protein (21, 23); thus, S protein might be a potential ligand for CS-GRP78 (Fig .1) (20). Treatment of cells with AR12, resulted in induction of GRP78 degredation and suppression of production of infectious virions via autophagosome formation. This treatment reduced viral entry through GRP78 (24).

## GRP78 vs. ACE2 in SARS-CoV2 clonization

Several studies have highlighted CS-GRP78 as a receptor for different viruses (25-27). Apart from DPP4 (CD26), which was shown to be the main receptor for MERS-CoV infection (28), it has been shown that CS-GRP78 facilitate viral entry into the host cells by sustaining viral attachment (29) and plays a crucial role in this process (30). Based on various sign and symptoms in COVID-19 patients, many researchers have suggested that SARS-CoV-2 predominantly targets endothelial cells, one of the largest populations of cells in the human body (31). GRP78 is broadly expressed in all endothelial cells, but it is upregulated in specific circumstances such as cancer. Owing to the this upregulation, it can be assumed that cancer patients are at higher risk for COVID-19 and severe complications (32).

GRP78 also presents certain properties that can make it a predominant receptor over ACE2 for SARS-CoV-2. Many tissues express only one pairs of ACE2/TMPRSS2 complex (15). While the ACE2 requires association of TMPRSS2 to cleave the S protein (9-11), the ABD domain at the N-terminus of GRP78 can simultanously provide the energy required for the successful entry of SARS-CoV-2 (33). Therefore, researchers assumed that CS-GRP78 could be an alternative receptor for SARS-CoV-2 and suggested natural and synthetic GRP78 inhibitors to block virus entry. For instance, Palmeira and colleagues by in silico analysis identified 409 compounds that can block the binding of the S protein to CS-GRP78 (30). In addition, Sudeep and colleagues reported optimal interaction features of Withaferin A, curcumin and andrographolide, natural ligands for the GRP78 receptor to block virus clonization (34).

All toghether, SBD is necessary for binding to the S protein and ABD provides required energy. Both domains of GRP78 are required for the entry of viruses such as EBOV (35), Borna Disease virus (25), MERS (28), and COVID-19 (30). The cited papers provided details of the function.

## Closing remarks

Although several reports have proposed GRP78 and other receptors as possible receptor for SARS-CoV-2 based on in silico analysis and a few experiments, there is no comprehensive documented paper in which the related data in this subject have been collected, discussed, and the evidences analyzed so far. The concept of existing an alternative receptor for virus entry can explain the involvement of different organs with very low expression of ACE2. This idea will be beneficial for readers to understand that why there is no strong association between the abundance of ACE2/TMPRSS2 co-expression and clinical features of the disease and the severity of complications. However, we provided additional data in terms of the mechanism of entry and function of the receptors. On the other hand, we reviewed other papers that suggested other receptors for SARS-CoV-2 entry and colonization, however our focus in this paper is on GRP78 as an alternative receptor. This protein is very common in different cells and a minor stress can activate this pathway and provide appropriate condition for virus entry.

In summary, the potential role of GRP78 in SARS-CoV-2 entry to the host cells convinced us to suggest that CS-GRP78 can be considered as an alternative receptor for this virus. Further experiments are recommended to confirm this idea.
